# The Need for Standardization of PRAME Immunohistochemistry in Melanocytic Neoplasms

**DOI:** 10.3390/dermatopathology13010005

**Published:** 2025-12-31

**Authors:** Calla M. Sullivan, Dominick DiMaio, Scott Lauer, Dinesh Pradhan, Julie Youngs, Kaeli Samson, Corey J. Georgesen

**Affiliations:** 1 Department of Dermatology, University of Nebraska Medical Center, Omaha, NE 68198, USA; casullivan@unmc.edu; 2 College of Medicine, University of Nebraska Medical Center, Omaha, NE 68198, USA; 3 Department of Pathology, Microbiology, and Immunology, University of Nebraska Medical Center, Omaha, NE 68198, USA; ddimaio@unmc.edu (D.D.); scott.lauer@unmc.edu (S.L.); dpradhan@unmc.edu (D.P.); jyoungs@unmc.edu (J.Y.); 4 Department of Biostatistics, University of Nebraska Medical Center, Omaha, NE 68198, USA; kksamson@unmc.edu

**Keywords:** PRAME, immunohistochemistry, melanoma, nevus

## Abstract

Accurate diagnosis of melanomas is crucial for proper evaluation and treatment. One immunohistochemical stain frequently utilized is PRAME (PReferentially expressed Antigen in MElanoma), a tumor-associated antigen expressed in most melanomas. This study aims to evaluate the reproducibility of PRAME scoring performed by dermatopathologists at an academic tertiary referral medical center. A blinded survey was designed featuring 21 melanocytic neoplasms stained with PRAME and H&E. For each case, five dermatopathologists provided a PRAME score from 0–4+, percent PRAME positivity, values for H-score, and a descriptive interpretation. Absolute agreement across raters was assessed using a Kappa statistic for PRAME score and intraclass correlations (ICCs) for H-score and PRAME percentage. Statistical analysis indicated poor inter-rater reliability for PRAME score (Kappa = 0.16), percent PRAME positivity (ICC = 0.31), and H-score (ICC = 0.40). Reporting language varied among pathologists. Our study demonstrated that the interpretation of PRAME immunohistochemistry lacks reproducibility, especially for challenging lesions. This suggests that a more rigorous, defined, and reproducible scoring method should be investigated for equivocal cases. Future studies may explore the utility of artificial intelligence software in the interpretation of PRAME for borderline lesions to improve reliability and standardize scoring.

## Simple Summary

Accurate diagnosis of melanoma, a skin cancer originating from melanocytes, is crucial for the appropriate treatment of patients. Pathologists often use a laboratory stain called PRAME (PReferentially expressed Antigen in MElanoma) to help distinguish melanoma from benign skin lesions, but interpretation can be challenging. In this study, five dermatopathologists reviewed twenty-one borderline melanocytic skin lesions to evaluate the consistency of PRAME interpretation. We found that PRAME scoring lacks reproducibility among dermatopathologists for these challenging lesions. This variability highlights the need for more rigorous and standardized scoring methods in order to improve diagnostic accuracy. Future studies should explore whether artificial intelligence-based tools can help improve reliability.

## 1. Introduction

Melanoma, the malignant transformation of melanocytes, can develop from existing precursor lesions or healthy-appearing skin and is associated with sun exposure, genetic factors, and older age [1]. The incidence of malignant melanoma is increasing rapidly worldwide, accounting for 4–5% of all newly diagnosed cancers and the fifth-highest number of lives lost across all cancers. The five-year survival rate for patients with stage I melanoma is over 97%, while the survival rate for those with stage IV disease is only about 30% [1].

Therefore, timely and accurate diagnosis of melanoma is critical for effective evaluation and treatment. Immunohistochemistry has recently become more prevalent in the evaluation of equivocal melanocytic lesions [2]. One immunohistochemical stain of recent increased utilization is PRAME (PReferentially expressed Antigen in MElanoma), a tumor-associated antigen originally isolated from a melanoma patient expressed in most melanomas [3]. Studies have confirmed PRAME as a valuable tool; it was initially shown that diffuse PRAME expression is present in 90% of conventional melanomas and that increasing dysplasia of melanocytic nevi corresponds with greater PRAME expression [3,4]. However, limitations include low frequency of expression in desmoplastic melanomas, rare positive expression in benign melanocytic nevi, lack of diffuse positivity in all melanomas, and subjectivity of interpretation in samples when 50–75% of cells are labeled [5]. Previous studies have highlighted challenges in the diagnosis of difficult melanocytic lesions and discordance between expert pathologists, but prospective studies performed amongst blinded dermatopathologists are lacking [6]. Thus, while PRAME remains diagnostically useful, the reproducibility among pathologists must be further investigated, and scoring techniques should be better defined, fine-tuned, and widely accepted in standard practice.

The primary objective of this study was to evaluate the reliability and reproducibility of PRAME immunohistochemical scoring of melanomas and benign melanocytic neoplasms as evaluated by dermatopathologists at a tertiary medical center. We seek to highlight that a definitive, reproducible, unequivocal grading system has yet to exist, and we will analyze current trends and provide suggestions for refining the precision of immunohistochemical grading in routine practice.

## 2. Materials and Methods

A blinded survey was created featuring 21 digital PRAME-stained whole slide images and corresponding Hematoxylin and Eosin (H&E)-stained slides. Samples were randomly selected from biopsies performed at a tertiary referral care center from 2021 to 2023 with PRAME immunohistochemistry ordered at the time of biopsy. No patient characteristics were recorded. Digital slides were not standardized for brightness or color. At our institution, PRAME is performed on borderline or challenging lesions; therefore, self-selection for diagnostic difficulty is assumed. PRAME IHC was performed using the BenchMark ULTRA instrument (Ventana Medical Systems, Tucson, AZ, USA). DAB chromogen and antibody clone EPR20330 were utilized; CC1 solution was pretreated for 32 min, and ready-to-use primary antibody was incubated for 32 min (Ventana Medical Systems, Tucson, AZ, USA). Metastatic melanoma (to the skin) was used as a positive control.

The samples were previously interpreted using PRAME IHC and included 11 benign nevi, 5 melanomas in situ, and 5 malignant melanomas. The benign nevi consisted of borderline atypical/dysplastic nevi, and no special classes, such as Spitz or blue, were included. No cases of melanoma arose in association with a nevus. Five board-certified dermatopathologists were provided with H&E-stained and PRAME-stained slides for each case. The slides were randomly ordered to create the final survey, and all dermatopathologists had the same order of questions. Raters were asked to independently analyze the slides using the pace and methods of their regular practice. Participating dermatopathologists included a mix of junior and senior full-time faculty who have practiced for a range of 2–24 years. All have completed a dermatopathology fellowship with either dermatology or pathology residency training.

For each of the 21 biopsies, survey participants provided a score from 0 to 4+, an estimated percentage of PRAME score, values to calculate an H-score, and an overall impression of positive or negative (with descriptive language allowed) for melanoma. The PRAME score of 0–4+ was based on the percentage of cells with positive staining for PRAME [3]. A score of 0 indicated no staining, 1+ was 1–25%, 2+ was 26–50%, 3+ was 51–75%, and 4+ indicated that 76% or more of the cells were positive [3]. The percent PRAME positivity that correlated with the 0–4+ PRAME score was also reported. Participants also provided values to calculate an H-score, which integrates both staining intensity and the proportion of positive cells. The percentage of cells that were negative (0), weakly stained (1+), moderately stained (2+), or strongly stained (3+) was reported. An H-score was then calculated using the following formula: (1 × percentage of 1+ cells) + (2 × percentage of 2+ cells) + (3 × percentage of 3+ cells) [7]. H-scores range from 0–300, with higher numbers representing stronger and more diffuse staining. Finally, pathologists gave an overall impression of PRAME results using the descriptive language they typically use in their reports.

For the ordinal measure (i.e., PRAME scores), exact agreement across the five raters was assessed using a Kappa value derived from a cumulative probit generalized linear mixed model, with random effects for raters and items. For continuous measures (i.e., H-scores and PRAME percentage), absolute agreement across the five raters was assessed using intraclass correlations (ICCs) and associated 95% confidence intervals derived from two-way random effects models, using the methodology of Shrout and Fleiss (1979) [8]. All analyses were performed using SAS software version 9.4 (SAS Institute Inc., Cary, NC, USA).

## 3. Results

Statistical analysis of PRAME score produced a Kappa result equal to 0.16 (95% CI: 0.09–0.23), which can be interpreted as “none to slight” interrater reliability, defined as a Kappa < 0.2 on a 1 to 1 scale [9]. Intraclass correlation coefficients calculated for percent PRAME positivity and H-score were equal to 0.31 (95% CI: 0.13–0.54) and 0.40 (95% CI: 0.21–0.63), respectively, indicating “poor” reliability, which is defined as an ICC < 0.5 on a 0 to 1 scale [10]. Heat maps were created to visualize patterns for all three measures, and the trends appear generally similar (Table 1, Table 2 and Table 3).

A PRAME score of 4+ is interpreted as positive, as described in Lezcano et al. [3]. The number of 4+ scores reported by each pathologist ranged between three to eight, with a total of eight, six, four, three, and eight positive results given by pathologists A–E, respectively. Pathologist A never gave a score of 1+, whereas pathologists D and E scored ten cases 1+. Pathologist E only gave three total scores of 2+ or 3+, meaning they largely graded on the extremes. Pathologists B and C both gave wider distributions of scores from 1–4+.

Of the twenty-one cases, fourteen received at least one positive result, and of those fourteen, seven received a 1+ score from another pathologist. Of the five cases originally diagnosed as melanoma, four were given at least one 4+ score. Furthermore, only three cases received a unanimous 4+ positive result; the original diagnoses of these were melanoma (case 3), melanoma in situ (case 13), and nevus (case 21).

Dermatopathologists also varied in the language used to report an overall impression (Table 4).

Pathologists A and E generally used binary language, stating either “positive” or “negative” for most samples. All pathologists except E used further descriptive language for some samples to indicate specific localizations, patchiness, or diffusivity of staining. Examples of this include impressions such as “positive in scattered lesional cells,” “weak, patchy staining in a minor subset of lesional melanocytes (<75%),” and “focal non-diffuse.” Pathologist B reported most samples as either “patchy” or “positive” and never directly used the word “negative.” Pathologist C also did not state “negative” but rather described such samples as “non-diffuse” and called positive results “diffuse strong positives.” Pathologist D gave the longest descriptions, explaining strength, location, and positivity for almost all cases; this can be contrasted to pathologist E, who only gave one-word impressions of “positive,” “negative,” or “indeterminate.”

## 4. Discussion

This study suggests that though PRAME immunohistochemistry is a valuable tool for melanoma diagnosis, current grading practices lack interrater reliability, especially for challenging lesions. Differences in PRAME interpretation may be further compounded by variations in the language used by dermatopathologists to report overall impressions. PRAME score, percentage PRAME positivity, and H-score were all shown to have no or poor reliability.

Dermatopathologists vary in the grading of PRAME immunohistochemistry of challenging melanocytic lesions. Perhaps, the human eye is not adept at assessing quantitative percentage cutoffs when assessing immunohistochemical grading, even though computer-aided methods have been shown to be similar in quantifying IHC staining intensity [11]. This can be further complicated by the fact that implicit bias exists, in that dermatopathologists may seek to use PRAME immunohistochemistry to support their initial impressions on H&E and subconsciously skew their scoring accordingly. Though PRAME has particular use in specific circumstances (e.g., highlighting melanoma arising within a nevus in a melanocytic lesion with two cell populations), it harbors the potential pitfall of use for biased confirmation of the diagnostician’s impression on H&E. That is, the human eye may seek and manifest positivity when melanoma is the favored diagnosis on the initial impression. The presence of endogenous melanin pigment may further obscure analysis. Moreover, while the need for a quantitative measure exists for reproducibility, the creation of an arbitrary cutoff point does fail to encapsulate the nuances of what is likely a biologic spectrum in terms of PRAME expression.

Beyond a lack of reliability in scoring systems, analysis of the reporting language used revealed further discrepancies among dermatopathologists. Not all pathologists used simple “positive” and “negative” terminology, opting for language such as “non-diffuse” and “diffuse,” and most used longer phrases. Though further descriptive language can be explained by the challenging nature of the cases, the supplemental diagnostic tool is negated when consistency and accuracy are forfeited [12].

Previous studies have also demonstrated challenges with PRAME immunohistochemistry, especially for challenging or borderline lesions. Though diffuse positive staining is highly prevalent in conventional malignant melanomas, this can be seen in only about half of challenging borderline lesions and one in ten dysplastic nevi [6]. Samples with intermediate staining (between 50 and 75% positivity) and/or difficult features limit interpretation and reproducibility. One recent suggestion to increase the sensitivity and specificity of PRAME is to decrease the threshold of positivity to at least 3+, if not 2+ [13]. Though lower cutoffs may be less accurate, one study of 85 lesions found that 76.09% of melanomas were PRAME-positive and 84.61% of nevi were negative, with 2+–4+ being positive [14]. Previous studies have limitations such as pre-screening of cases or a lack of focus on borderline lesions, where diagnostic accuracy is crucial. Nonetheless, for these cases, PRAME immunohistochemistry requires correlation with adjunct immunohistochemistry, relevant clinical findings, and occasionally molecular evaluation and expert dermatopathologist consensus [5].

Furthermore, standardized scoring systems exist for other immunohistochemical stains that demonstrate interpretive variability. For example, ER/PR guidelines use cutoffs of <1% (negative), 1–10%, 10–50%, and >50%; pathologists report intensity, and standardized examples for each score are provided to help minimize discrepancies [15,16]. Though PRAME has more limited sensitivity and specificity, a similar, more rigorous and reproducible scoring method should be investigated for PRAME, especially for equivocal cases. Another potential way to increase standardization would be to include a melanocyte marker (such as Sox-10 or Melan-A) for comparison. A limitation of our study was that not all cases had this immunohistochemistry, and it may be unrealistic to order for every case. Still, melanocyte markers may allow for more accurate assessment of PRAME, although no rigorous standardization process exists for this scenario. Furthermore, increased clinical–pathological correlation represents another avenue to decrease diagnostic discordance by allowing for better correlation of clinical and histopathological features. Despite logistical challenges, the establishment of a multidisciplinary group has been shown to improve the accuracy of dermatology diagnoses [17].

**Figure 1:**
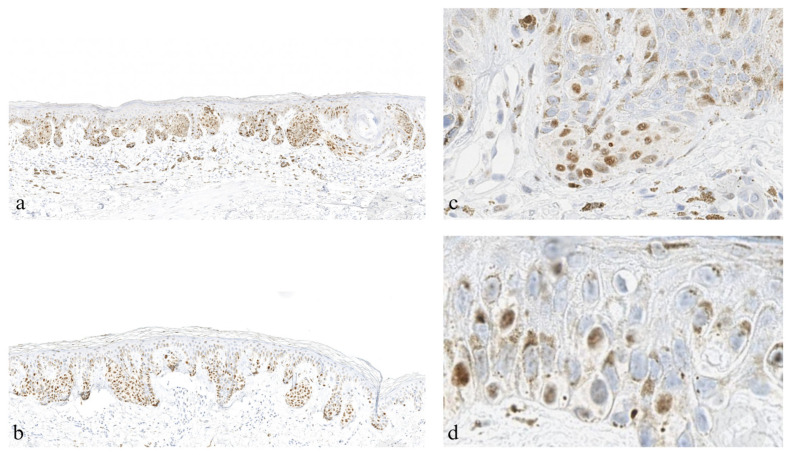
Melanoma samples stained with PRAME (PReferentially expressed Antigen in MElanom) demonstrating weak variable staining patterns despite strong positive controls. Magnification of 10× in panels (**a**,**b**), ~20× in panel (**c**), and ~40× in panel (**d**).

**Figure 2:**
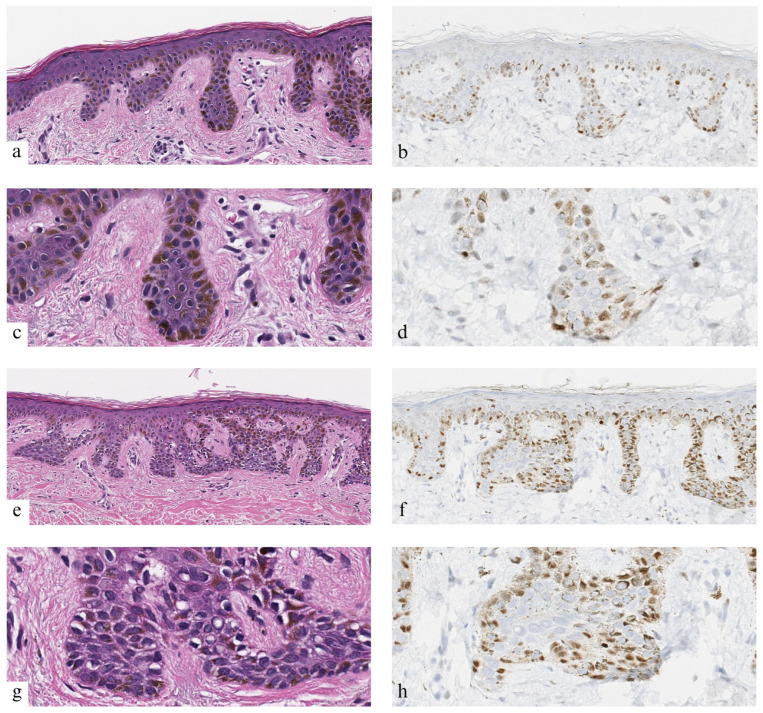
Representative examples of challenging lesions with discordant PRAME interpretations, Case 1 (**a**–**d**) and Case 12 (**e**–**h**). Panels (**a**,**c**,**e**,**g**) are stained with H&E; panels (**b**,**d**,**f**,**h**) show PRAME (PReferentially expressed Antigen in MElanom) immunohistochemistry; 10× magnification in panels (**a**,**b**,**e**,**f**); 20× in panels (**c**,**d**,**g**,**h**).

Though PRAME analysis is typically performed using the 0–4+ scoring system [3], the H-score (Histo-score) system may also provide an alternative or adjunct for cases with 50–75% staining. H-score considers the strength and distribution of a given stain and has been shown to be slightly superior compared to overall percentage positive scores [18]. In our experience, in some cases, PRAME exhibits weak staining of melanocytes (despite strong positive controls), and guidance is lacking when interpreting the strength of stain in the original PRAME 0–4+ scoring system (Figure 1 and Figure 2). This certainly contributes to inter-rater discordance, as a standard in the setting of weak staining has yet to be established. The H-score system has previously been applied to melanoma, for example, in assessing VISTA expression in the context of progression, further illustrating its potential applicability for PRAME IHC [19].

Our study also showed that H-score calculation may be more reliable; the ICC for H-score was slightly higher than percent PRAME positivity, which is a proxy for PRAME score. Still, both PRAME score and H-score have an apparent lack of interrater reliability. Further study is needed to apply H-score (and other scoring algorithms) to PRAME analysis, and this could potentially provide a supplemental tool for borderline cases.

However, H-score takes longer to calculate, especially if done manually, and it requires established cutoffs for negative, weak, moderate, or strongly positive staining [18]. Online calculators or artificial intelligence image recognition software could potentially improve the speed and convenience of H-score; still, AI solutions present drawbacks, including the need for large training datasets and algorithm validation, potential biases, and ethical and regulatory challenges [20]. Still, deep learning models have been preliminarily implemented for the calculation of the H-score [7]. Along with other modalities such as genetic sequencing, standardized AI scoring platforms in the age of digital pathology could improve precision, accuracy, and reliability when utilizing PRAME as a supplement to melanoma diagnosis.

In summary, this study evaluated the reproducibility of PRAME immunohistochemistry scoring of 21 melanocytic lesions performed by dermatopathologists at a single institution and showed little to no reproducibility for PRAME score, percent overall positivity, and H-score in equivocal cases. This study reflects useful preliminary observations, but further study is required to support more definitive statements on PRAME reproducibility. Limitations of this study include (1) a small sample size of melanocytic lesions was used, which may limit the stability of statistical analysis, (2) the study was performed among dermatopathologists within a single institution with varying lengths of clinical practice, (3) the specific inclusion of borderline/difficult diagnostic cases, with the assumption of difficulty based on PRAME immunohistochemistry having been previously performed, (4) the use of digital DAB images without additional melanocytic markers, as endogenous melanin may interfere with interpretation, (5) digitization of slides could lead to variation in perception of staining, and (6) specific nevus subtypes were excluded. Future studies should include larger datasets, involve multiple institutions, incorporate additional melanocytic markers or chromogens (e.g., red carbazole), and apply H-score (and/or other scoring methods) to PRAME. Ultimately, the use of artificial intelligence scoring algorithms may serve as a boon to improve reliability and standardize PRAME interpretation.

## Figures and Tables

**Table 1:** PRAME score heat map and original pathology diagnosis. Darker shades of red correspond to increased PRAME score.

	Path A	Path B	Path C	Path D	Path E	Average	Range	Original Dx
Case 1	4	1	3	2	2	2.4	3	Melanoma in situ
Case 2	3	2	2	2	3	2.4	1	Melanoma in situ
Case 3	4	4	4	4	4	4	0	Melanoma
Case 4	2	2	2	1	1	1.6	1	Nevus
Case 5	2	3	1	1	3	2	2	Nevus
Case 6	3	2	3	2	4	2.8	2	Nevus
Case 7	2	2	3	1	1	1.8	2	Melanoma in situ
Case 8	4	4	3	2	1	2.8	3	Nevus
Case 9	2	3	4	2	4	3	2	Melanoma in situ
Case 10	2	3	2	2	4	2.6	2	Nevus
Case 11	2	3	2	1	4	2.4	3	Melanoma
Case 12	4	2	1	2	1	2	3	Melanoma
Case 13	4	4	4	4	4	4	0	Melanoma in situ
Case 14	3	4	2	1	1	2.2	3	Nevus
Case 15	2	4	3	3	4	3.2	2	Melanoma
Case 16	4	2	3	1	1	2.2	3	Nevus
Case 17	2	1	2	1	1	1.4	1	Nevus
Case 18	4	1	1	1	1	1.6	3	Nevus
Case 19	3	1	3	1	1	1.8	2	Nevus
Case 20	3	1	3	1	1	1.8	2	Melanoma
Case 21	4	4	4	4	4	4	0	Nevus

**Table 2:** Overall percent positivity for PRAME heat map. Darker shades of red correspond to increased percent positivity.

	Path A	Path B	Path C	Path D	Path E	Average	Range
Case 1	80	20	65	40	40	49	60
Case 2	60	50	40	30	70	50	40
Case 3	80	80	95	80	95	86	15
Case 4	70	30	40	20	10	34	60
Case 5	60	65	20	20	60	45	45
Case 6	70	40	70	50	90	64	50
Case 7	60	45	65	20	10	40	55
Case 8	80	75	65	40	20	56	60
Case 9	40	65	95	40	90	66	55
Case 10	60	70	20	30	90	54	70
Case 11	0	55	25	30	90	40	90
Case 12	90	40	10	40	20	40	80
Case 13	80	95	95	90	95	91	15
Case 14	60	80	60	20	20	48	60
Case 15	60	75	65	60	90	70	30
Case 16	90	30	0	20	5	29	90
Case 17	50	15	50	5	5	25	45
Case 18	80	25	20	25	20	34	60
Case 19	70	20	75	10	25	40	65
Case 20	50	15	75	25	20	37	60
Case 21	80	80	95	80	85	84	15

**Table 3:** Calculated H-score heat map. Darker shades of red correspond to increased H-score.

	Path A	Path B	Path C	Path D	Path E	Average	Range
Case 1	190	60	190	90	110	128	130
Case 2	130	150	110	60	200	130	140
Case 3	210	240	285	210	290	247	80
Case 4	130	70	90	30	10	66	120
Case 5	130	145	35	30	170	102	140
Case 6	160	100	180	80	260	156	180
Case 7	160	125	115	42	20	92.4	140
Case 8	150	200	125	70	20	113	180
Case 9	110	190	280	60	260	180	220
Case 10	120	120	110	60	270	136	210
Case 11	110	165	75	75	270	139	195
Case 12	120	65	10	60	100	71	110
Case 13	220	285	290	270	285	270	70
Case 14	170	240	155	50	120	147	190
Case 15	150	225	195	150	270	198	120
Case 16	200	90	170	42	10	102.4	190
Case 17	140	45	170	10	10	75	160
Case 18	210	75	50	50	60	89	160
Case 19	170	60	225	50	75	116	175
Case 20	130	45	230	55	60	104	185
Case 21	230	240	290	230	255	249	60

**Table 4:** Descriptive language utilized in pathology reports. Language used to describe 4+ PRAME scores is highlighted in gray.

Descriptive Language	# of Uses	Pathologist(s)	PRAME Score(s)
Positive	21	A, B, E	4+
Diffuse strong positive	3	C	4+
Strongly and diffusely positive in lesional melanocytes	3	D	4+
Weakly positive in the majority of lesional cells	1	A	4+
Diffuse strong intensity positive staining in the junctional melanocytes; Negative in dermal melanocytes	1	C	4+
Negative	22	A, D, E	3+, 2+, 1+
Patchy	12	B	3+, 2+, 1+
Non-diffuse	6	C	3+, 2+
Positive in scattered lesional cells, interpreted as overall negative	5	A	3+, 2+
Indeterminate, or other	3	D, E	3+, 1+
Non-diffuse moderate to strong intensity staining in the majority of the junctional melanocytes	4	C	3+
Patchy (positive in epidermal)	2	B	3+
Diffuse positive strong intensity staining in the majority of the junctional melanocytes	1	C	3+
Strong intensity, near diffuse positive staining	1	C	3+
Weak, patchy staining in a minor subset of lesional melanocytes (<75%)	10	D	2+, 1+
Patchy positivity in a minor subset of lesional melanocytes (<75%)	4	D	2+, 1+
Strong positive in a subset of junctional melanocytes, weak to negative in dermal melanocytes, suggestive of MIS with nevus	2	C	2+
Very focally positive	1	B	1+
Focal non-diffuse	1	C	1+
Focal weak intensity staining (negative)	1	C	1+
(Non-diffuse) patchy staining in a minority of junctional melanocytes	1	C	1+

## Data Availability

The raw data supporting the conclusions of this article will be made available by the authors upon request.

## Abbreviations

The following abbreviations are used in this manuscript:

PRAME	Peeferentially Expressed Antigen in Melanoma
H&E	Hematoxylin and eosin
IHC	Immunohistochemistry
DAB	3,3′-Diaminobenzidine
CC1	Cell Conditioning 1
H-score	Histo-score
ICC	Intraclass correlation
ER/PR	Estrogen receptor/progesterone receptor
AI	Artificial intelligence
